# Applications of Bayesian Phylodynamic Methods in a Recent U.S. Porcine Reproductive and Respiratory Syndrome Virus Outbreak

**DOI:** 10.3389/fmicb.2016.00067

**Published:** 2016-02-02

**Authors:** Mohammad A. Alkhamis, Andres M. Perez, Michael P. Murtaugh, Xiong Wang, Robert B. Morrison

**Affiliations:** ^1^Department of Veterinary Population Medicine, College of Veterinary Medicine, University of MinnesotaSt. Paul, MN, USA; ^2^Environmental and Life Sciences Research Center, Kuwait Institute for Scientific ResearchKuwait City, Kuwait; ^3^Department of Veterinary and Biomedical Sciences, University of MinnesotaSt. Paul, MN, USA

**Keywords:** Bayesian phylodynamics, PRRSV, RFLP type 1-7-4, ORF5 gene, molecular surveillance

## Abstract

Classical phylogenetic methods such as neighbor-joining or maximum likelihood trees, provide limited inferences about the evolution of important pathogens and ignore important evolutionary parameters and uncertainties, which in turn limits decision making related to surveillance, control, and prevention resources. Bayesian phylodynamic models have recently been used to test research hypotheses related to evolution of infectious agents. However, few studies have attempted to model the evolutionary dynamics of porcine reproductive and respiratory syndrome virus (PRRSV) and, to the authors' knowledge, no attempt has been made to use large volumes of routinely collected data, sometimes referred to as big data, in the context of animal disease surveillance. The objective of this study was to explore and discuss the applications of Bayesian phylodynamic methods for modeling the evolution and spread of a notable 1-7-4 RFLP-type PRRSV between 2014 and 2015. A convenience sample of 288 ORF5 sequences was collected from 5 swine production systems in the United States between September 2003 and March 2015. Using coalescence and discrete trait phylodynamic models, we were able to infer population growth and demographic history of the virus, identified the most likely ancestral system (root state posterior probability = 0.95) and revealed significant dispersal routes (Bayes factor > 6) of viral exchange among systems. Results indicate that currently circulating viruses are evolving rapidly, and show a higher level of relative genetic diversity over time, when compared to earlier relatives. Biological soundness of model results is supported by the finding that sow farms were responsible for PRRSV spread within the systems. Such results cannot be obtained by traditional phylogenetic methods, and therefore, our results provide a methodological framework for molecular epidemiological modeling of new PRRSV outbreaks and demonstrate the prospects of phylodynamic models to inform decision-making processes for routine surveillance and, ultimately, to support prevention and control of food animal disease at local and regional scales.

## Introduction

Porcine Reproductive and Respiratory Syndrome (PRRS) is, arguably, the most important swine disease in the United States due to the continuous emergence of new outbreaks that cause severe economic losses (Neumann et al., 2005; Holtkamp et al., 2013). Type 2 PRRSV, which is endemic in North America, was discovered in 1989 in the U.S., although the earliest serological evidence was found in eastern Canada (Benfield et al., 1992; Zimmerman, 2003; Murtaugh et al., 2010). PRRSV is a single-stranded, enveloped RNA virus that belongs to the *Arteriviridae* family (Benfield et al., 1992). Its genome consists of nine open reading frames (ORF) that code seven structural proteins and 14 non-structural proteins (Dokland, 2010). ORF5 encodes a major envelope surface glycoprotein (GP5) with high genetic diversity, thus has been widely used in molecular epidemiology studies of PRRSV (Kapur et al., 1996; Shi et al., 2010; Brar et al., 2015).

PRRSV transmission is rapid and can occur through direct and indirect contact (Dea et al., 2000; Cho et al., 2007). Emerging PRRSV strains are capable of spreading over long distances, referred to as distance-independent dispersal, as a result of aerosol transmission, animal movements, and use or movement of contaminated semen, equipment, or trucks (Shi et al., 2010, 2013). The combination of varied transmission routes and absence of regulated control and prevention activities makes virus control or elimination, at both local and regional levels, extremely challenging (Corzo et al., 2010; Rowland and Morrison, 2012). Hence, intensifying efforts toward designing effective and efficient surveillance programs, with the long-term goal of eliminating the disease, must be prioritized to minimize the current impact of the PRRSV on the US swine industry (Perez et al., 2015).

Since the 1980's, the U.S. Department of Agriculture has conducted extensive surveillance activities for swine diseases using classical statistical sampling methods that can account for imperfect diagnostic testing (Cameron and Baldock, 1998). However, current disease surveillance activities do not fully account for modern swine production systems in which pigs are spatially separated by age or production stage, or for pathogens that evolve rapidly (Rowland and Morrison, 2012; Perez et al., 2015).

In the past few decades, many studies investigated the molecular epidemiology of PRRSV, due to its high potential for mutation and recombination (Martín-Valls et al., 2014). Some studies focused on establishing associations between the evolutionary features of PRRSV and epidemiological characteristics of outbreaks in different geographical levels (Goldberg et al., 2000; Shi et al., 2010, 2013; Yoon et al., 2013; Nguyen et al., 2014; Rosendal et al., 2014). Others discriminated between novel and preexisting strains to model viral spread and maintenance within affected populations (Larochelle et al., 2003; Tun et al., 2011; Alonso et al., 2013; Brito et al., 2014; Chen et al., 2015). Whether the studies used classical phylogenetic methods to either genotype newly emerging PRRSV strains on the basis of restriction fragment length polymorphism (RFLP) patterns, or assessed correlations between the similarities of nucleotide sequences and other epidemiologic features, they typically ignored uncertainties associated with estimates of phylogenetic relationships, temporal factors, and spatial factors (Suchard et al., 2001). Furthermore, they examined the temporal and spatial dynamics of the virus isolates in separate methodological settings, and attempted to draw conclusions from the outputs of both epidemiological and evolutionary analytical methods (Suchard et al., 2001). Therefore, many methodological approaches previously used to study PRRSV have ignored that evolutionary and epidemiological dynamics of rapidly evolving pathogens like PRRSV occur on approximately the same time-scale, and thus, they must be studied in a unified methodological setting in order to be properly understood and to prevent biased conclusions, subsequently improving the related decision making processes (Pybus et al., 2013). The field of phylodynamics aims to model, in a Bayesian statistical framework, the joint evolutionary, and epidemiological characteristics of rapidly evolving pathogens using analytical methods from the well-established field of phylogenetics (Grenfell et al., 2004). This approach uses important evolutionary parameters of rapidly evolving pathogens as random variables, and assigns a specified prior probability distribution for each parameter to infer their corresponding posterior probability distribution (Lemey et al., 2009). Thus, such Bayesian framework provides powerful analytical tools capable of accounting for uncertainties in the evolutionary parameters, including the pathogen phylogeny, population demographics, size, and history of dispersal between geographical regions and hosts (Lemey et al., 2009).

Bayesian phylodynamic models have recently become well-established tools for studying the evolution of many infectious viral diseases. However, only a few studies have modeled the evolutionary dynamics of PRRSV (Tun et al., 2011; Shi et al., 2013; Brito et al., 2014; Nguyen et al., 2014; Chaikhumwang et al., 2015). Such studies revealed the potential of phylodynamic methods in answering many long-standing questions on the molecular epidemiology and evolution of PRRSV. Furthermore, the method has previously been applied in a research context, rather than for routine surveillance of field data intended to support disease prevention and control. Such implementation is challenging because of the complexity and size of the data being analyzed. Data with these features, sometimes referred to as big data, requires special procedures for preparation and analysis.

The objective of this study was to demonstrate the application of Bayesian phylodynamic models to data routinely collected by swine production systems to support a near real-time early warning surveillance system for PRRSV and, potentially, other food animal viruses. The method was applied to the spread of a virulent RFLP 1-7-4 type PRRSV between 2014 and 2015 in the U.S. A discrete-trait phylodynamic model was adopted to estimate both the geographical history of viral migration and the movement of the virus among age groups of pigs. Our study provides quantitative estimates of mechanisms that lead to the emergence, spread and maintenance of the RFLP 1-7-4 PRRSV family throughout the U.S. It further illustrates the prospects of the Bayesian approach in improving the decision making process related to reducing the impact of PRRS on the national swine industry with the long-term goal of successful control and prevention.

## Materials and methods

### Sequence data

Complete PRRS ORF5 nucleotide sequences (*n* = 6774) from field isolates obtained between January 1998 and April 2015 were provided by five independent swine production systems in the U.S. with metadata on the date of isolation, system code (A, B, C, D, and E) and type of farm (farrow to wean or farrow to feeder sow farms and growing pig farms) from which the sequences were isolated (Table S1). Sequences were deposited in Genbank with accession numbers KT902023–KT905410 and KU501407–KU504248. The data were shared under agreement that identity and location of participants and their respective farms was confidential. Sequencing was performed according to the procedures in use at the time in various veterinary diagnostic laboratories or in private laboratories on a fee-for-service basis.

### Preliminary phylogenetic analysis

The complete sequence database was manually validated for presence of a complete ORF5 and absence of ambiguous nucleotides then aligned using MUSCLE version 3.8 (Edgar, 2004). A maximum likelihood (ML) phylogenetic analysis was performed in MEGA6, resulting in identification of a cluster of 288 sequences that were further studied. The sequence file was re-aligned using MUSCLE, and adjusted manually using amino-acid translation method implemented in Mesquite version 3.01 (Maddison and Maddison, 2011), to ensure that the protein-coding region of ORF5 remained in frame. Sequences with 100% nucleotide identity were removed (34%) from the subsequent analyses. While using Recombination Detection Program version 3 (RDP3), no homologous recombination was detected in the remaining sequences (Martin et al., 2010). For this analytical approach, it is important to select the substitution model that best describes the specific virus. For example, it was found that for some Dengue viruses, the mixed substitution model best fit the data (Drummond and Rambaut, 2007). That may, however, not be true for PRRSV. Thus, the best fitting partitioning scheme and nucleotide substitution model were selected using the Bayesian Information Criterion (BIC) implemented in PartitionFinder V 1.1 (Lanfear et al., 2012). Finally, maximum-likelihood estimates of the phylogeny under the selected mixed-substitution model were used to assess the degree of topological (in)congruence, in which 100 non-parametric bootstrap replicate searches were performed using RAxML version 8 (Stamatakis, 2014).

### Divergence-time, growth rate, and population size estimation

Divergence time was estimated using the relaxed molecular-clock model with GTR+Γ_4_ mixed-substitution, which was selected based on the results of PartitionFinder analysis mentioned above, implemented in BEAST v 1.8 (Drummond and Rambaut, 2007). To estimate divergence time and viral growth rate within each production system, we assessed the fit of the sequence data to five node-age coalescent priors, namely, (1) constant population size assuming that the population growth rate is zero (Griffiths and Tavare, 1994); (2) exponential growth assuming that the population growth rate is fixed over time (Griffiths and Tavare, 1994); (3) expansion growth assuming that the population growth rate increases over time (Griffiths and Tavare, 1994); (4) logistic growth assuming that the population growth rate decreases over time (Griffiths and Tavare, 1994); and (5) piece-wise-constant Bayesian skyline coalescent model (BS) assuming effective population size is experiencing episodic stepwise change over time (Drummond et al., 2005). For each node-age model, we compared the uncorrelated exponential (UCED) and the log-normal (UCLN) relaxed clock branch-rate prior models, to assess whether our sequence data had a substitution rate on adjacent branches that sampled from either shared exponential or log-normal distributions, respectively. Isolation dates of the sequences were used to calibrate divergence-time estimates. We first estimated the marginal likelihood for each of the 10 candidate phylodynamic models from the resulting posterior samples using the posterior simulation-based analog of Akaike's information criterion (AICM; Raftery et al., 2007), which were estimated using Tracer version 1.6 (Suchard et al., 2001; Rambaut et al., 2014). The AICMs and their Monte Carlo standard errors (SE) were calculated using 1000 replicates. Bayes factor (BF) comparisons indicated that the sequence data followed a population expansion growth with a UCED branch-rate model, which provided the best fit for ORF5 (BF > 25 for the log marginal likelihood) among parametric models (Table S2). However, the BF comparison was not significant when the expansion model was compared against the BS coalescent tree prior (Table S2). Hence, the BS coalescent tree prior model with a UCED branch-rate was used to estimate changes in the effective population size through time (File S2; Minin et al., 2008).

We used the Markov Chain Monte Carlo (MCMC) algorithms implemented in BEAST to estimate the joint posterior probability distributions of the model parameters. For each MCMC simulation, we run 3 × 10^8^ cycles, which was thinned by sampling every 10,000 cycles. Two replicate MCMC simulations were carried out to aid in assessing simulation performance. We used Tracer to evaluate convergence of each candidate model by estimating effective sample sizes (ESS) for each posterior parameter. Hence, our ESS evaluations suggested that the MCMC algorithms requires the removal of the first 10% of the samples (the “burn-in”) to provide reliable approximations of the posterior probability densities for each estimated parameter. We used Tree Annotator to summarize the posterior results in form of maximum clade credibility (MCC) trees. A BS plot was generated to infer effective population size (EPS) of the virus between 2001 and 2015, in which the EPS is defined as the relative genetic diversity (NeT), where Ne is the effective population size and T is the generation time (Minin et al., 2008).

### Estimation of viral dispersal history between regional systems

Geographical location was incorporated as described elsewhere (Lemey et al., 2009). Briefly, We reconstructed the phylogeny of the virus by incorporating discrete traits (i.e., systems), to describe the dispersal evolution of PRRSV epidemic among those selected systems. We used the continuous-time Markov model implemented in BEAST to model the dispersal history among systems as discrete states, which comprised a number of non-zero transition rates identified by a Bayesian stochastic search variable selection (BSSVS) approach (Lemey et al., 2009). Furthermore, we investigated directionality of the geographical dispersal of the virus among systems by assessing the fit of the data to two candidate discrete trait models (Table S2), including both symmetric and asymmetric models with irreversible and reversible transitions, respectively. Here, the symmetric model with irreversible transitions indicate that the directional spread of the virus between two systems (A → B or/and B → A) is insignificant, while the asymmetric model with reversible transition indicate that the directional spread between two systems (A → B or/and B → A) is significant. To reconstruct the history of viral migration between discrete system areas, we used the coalescent Gaussian Markov Random field (GMRF) Bayesian Skyride model as a prior on the node times in the tree and a mean-one exponential prior for the rate parameters of the candidate models, while we used the same remaining parameters described in the above analyses (e.g., substitution model, UCLN, and UCED branch-rate models). Similarly, we estimated the marginal-likelihoods in order to compute the BFs to select among the candidate models (e.g., UCLN symmetric vs. UCED Asymmetric; Table S2; File S3). We used FigTree version 1.4 (Rambaut, 2012) to plot the summarized MCC consensus tree with the root state posterior probabilities (RSPP) of systems areas. Here, the RSPP is defined as the posterior probability of transition from one discrete trait to another mapped onto the interior nodes of the phylogeny of the virus, in which a discrete trait with a high RSPP indicate that trait as the likely ancestral trait of the given phylogeny. Finally, we used SPREAD version 1.0.6 (Bielejec et al., 2011) to identify non-zero transition rates between discrete traits (i.e., significant dispersal routes among systems). We used a BF cutoff = 6 to assess the strength and significance of transition rates between discrete geographic system areas. Because actual centroids of the site locations were confidential, relationally correct, anonymous latitude and longitude locations were placed in Alaska and a keyhole markup language (KML) file was generated to visualize regional migration of the virus.

### Modeling viral transmission in a system

Evolutionary movement between farm types (a proxy for production type), in which farm type were classified as sow herd (e.g., farrow to wean and farrow to feeder sow), and all other farms (e.g., finisher and nursery). A discrete-trait model was used for farm type (sow herd, other farms) to infer the history of PRRSV migration between farm types through time. The number of non-zero transition rates in the model was estimated using BSSVS. The relative strength of transition rates (e.g., sow farms → all other farms) was estimated using Bayes factors (BFs). We estimated the ancestral states (farm type) at internal nodes of the tree under a composite phylogenetic model that included the above detailed analyses. We used FigTree to plot the MCC consensus tree with the RSPP of the discrete trait and we assessed the strength of transition rates between states (farm types) using the BF comparisons implemented in SPREAD similar to the above analyses. Similarly, the use of the asymmetric or symmetric discrete trait models allowed us to assess the strength and significance of directionality between farm types (e.g., sow farms → other farms, or/and farms → sow farms; File S4).

### Uncertainty and statistical analysis of discrete-trait mappings

We used the Kullback–Leibler divergence (KL) statistic to quantify the magnitude of phylogenetic uncertainty in the discrete-trait estimates of the RSPP (for regional systems and farm type; Kullback and Leibler, 1951). KL statistics were calculated for each selected tree using the Razavi function (Razavi, 2008) implemented in Matlab v 2013a (MathWorks, 2012) to measure the departure between prior and the corresponding posterior probability distributions for a given phylodynamic parameter (i.e., in this case the RSPPs). A large KL-value (KL > 1) indicates that the prior provided sufficient information for estimating the posterior parameters. Finally, we calculated the parsimony score (PS) and the association index (AI) statistics to assess the hypothesis that a taxon with a given trait (farm type or regional system) is more likely to share that trait with adjoining taxa in the MCC tree than would be expected by chance. The AI and PS statistics were calculated using Bayesian Tip-Significance Testing (BaTS) software version 1.0 (Parker, 2008). Significant AI and PS statistics indicate that our selected trait did have a significant role in shaping the posterior phylogeny of the sequence data.

## Results and discussion

### Preliminary phylogenetic analysis

**Figure 1:**
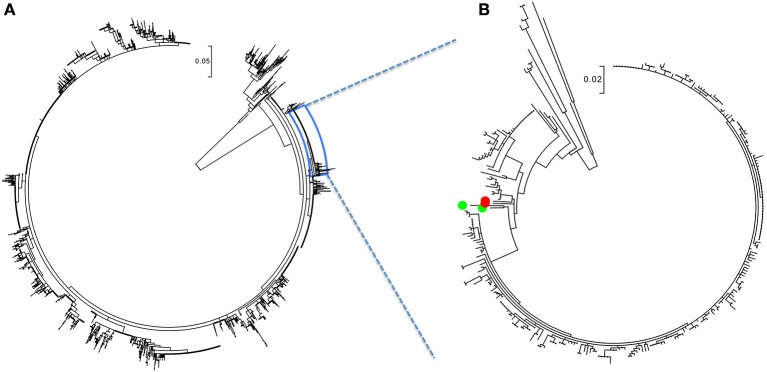
**PRRSV RFLP 1-7-4 cluster filtering. (A)** Maximum likelihood tree of complete PRRSV sequence dataset (*N* = 6774) with the 1-7-4 cluster expanded. **(B)** The ML tree of extracted sequences containing the 1-7-4 cluster. Two nearest neighbor 1-7-4 RFLP types (green dots) were collected in August 2012 and March 2007. Two red dots indicated a 1-7-4 type isolated in January 2004 and a 1-4-4 type isolated in October 2006. The figure was generated from File S1.

The ML analysis was performed to screen-out unrelated strains and because, although RFLP nomenclature is typically used to refer to PRRSV strains, the RFLP method is not an accurate discriminator of phylogenetic relations. As a result of the ML analysis, a total of 288 sequences, with isolation dates between September 2003 and March 2015, formed a phylogenetic branch shown in Figure 1. Within the branch a single, monophyletic clade of 241 sequences obtained in 15 months, between January 2014 and March 2015, stood out (Figure 1, Table S1). Those 241 were identified by the dominant RFLP-type, 1-7-4, whereas the other 45 genetically related strains belonged to a number of other RFLP types. Two nearest neighbor 1-7-4 RFLP types (depicted as green dots in Figure 1, Table S1) were collected in August 2012 and March 2007, whereas the two red dots indicated a 1-7-4 type isolated in January 2004 and a 1-4-4 type isolated in October 2006 (Figure 1, Table S1).

### Divergence-time, growth rate, and population size

**Figure 2:**
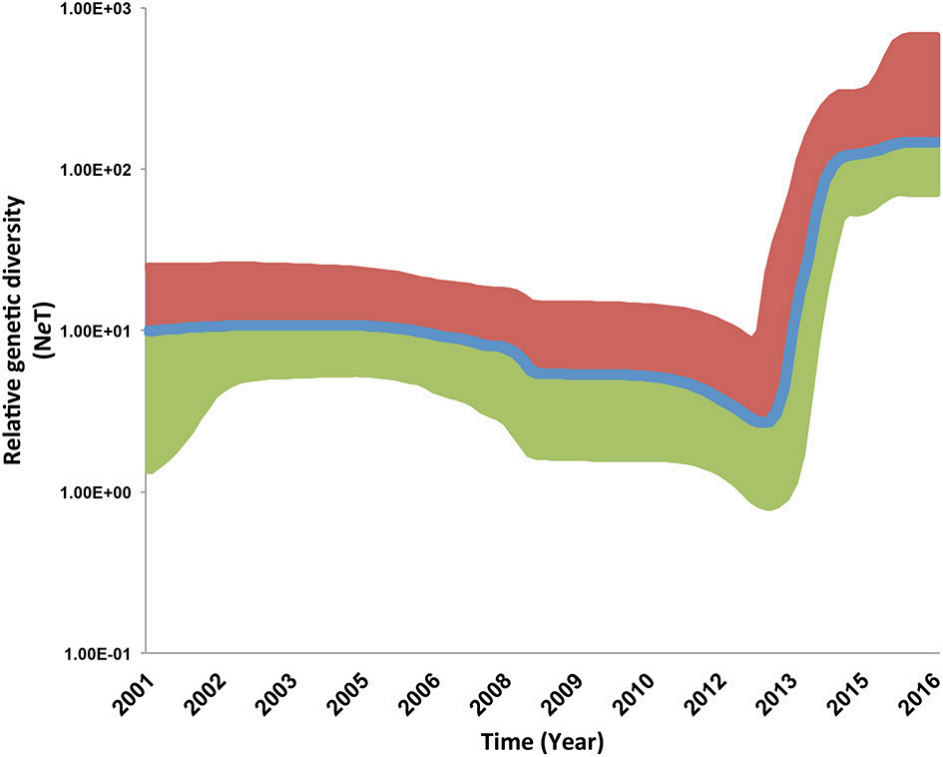
**Bayesian Skyline plots (BSP) illustrating temporal changes in the relative genetic diversity of Porcine Reproductive and Respiratory Syndrome Virus RFLP type 1-7-4 isolated between September 2003 and March 2015 in the United States estimated from the ORF5 gene sequences**. Line plots summarize estimates of the effective population size (*N*_*e*_*T*), a measure of genetic diversity, for ORF5 gene segment; the shaded regions correspond to the 95% HPD (Upper, Red; Median, Blue; Lower, Green).

For the PRRSV ORF5 sequence dataset isolated between September 2003 and March 2015, the BF comparisons significantly favored the parametric expansion node-age coalescent model, indicating that the population size of the current 1-7-4 type PRRSVs was under rapid increase with an estimated mean growth rate of 1.02 (95% highest posterior density, HPD, from 0.59 to 1.46) and mean evolutionary rate of 3.27 × 10^−3^/site/year (95% HPD from 2.37 × 10^−3^ to 4.27 × 10^−3^), which lays within the range of previously estimated evolutionary rates for PRRSVs isolated from different geographical locations and period of times (Forsberg, 2005; Nguyen et al., 2014; Chaikhumwang et al., 2015). However, analysis of the virus population dynamics revealed a distinct continuous increase in the genetic diversity of the virus in March 2015, with no signs of population decline. This corresponds to the current increase of PRRSV incidence throughout the regional production systems in the U.S. (Figure 2). Our findings suggest the rate, or speed, at which the number of PRRSVs in the population increased, sometimes referred to as growth rate, was higher, compared to earlier phylogenetic relatives. This higher growth rate may suggest expanding diversity, and an unusual continuous increase in the relative genetic diversity over time, compared to those earlier phylogenetic relatives. That finding may be attributed to an evolutionary drift that resulted from either continuous circulation or maintenance within the production region, or recombination events with field viruses migrated from other production regions (Wang et al., 2015). An earlier study also suggested that this expanding diversity behavior of newly emerging strains is attributed to environmental factors associated with the continuous changes in swine husbandry practices rather than intrinsic factors within the host species (Murtaugh et al., 2010). The estimated divergence time for this sequence dataset was September 1996 (95% HPD, July 1986–December 2001), which completely overlaps with the TMRCA of sequences isolated from system C (Table 1). The youngest divergence time estimated for the viruses isolated from system A was August 2009 (95% HPD, December 2007–August 2011; Table 1).

**Table 1:** **Estimated TMRCAs for the PRRSV ORF5 gene sequences isolated within each system**.

**System**	Mean TMRCA^*^	**Lower 95% HPD**	**Upper 95% HPD**
A	Aug-2009	Dec-2007	Aug-2011
B	Aug-2006	Nov-2003	Aug-2009
C	Sep-1996	Jul-1989	Dec-2001
D	Jul-2009	Jan-2007	Aug-2011
E	Jul-2009	Jan-2007	Aug-2011
Overall	Sep-1996	Jul-1989	Dec-2001

* *Time to the most recent common ancestor (TMRCA)*.

### Viral dispersal history between regional systems

**Figure 3:**
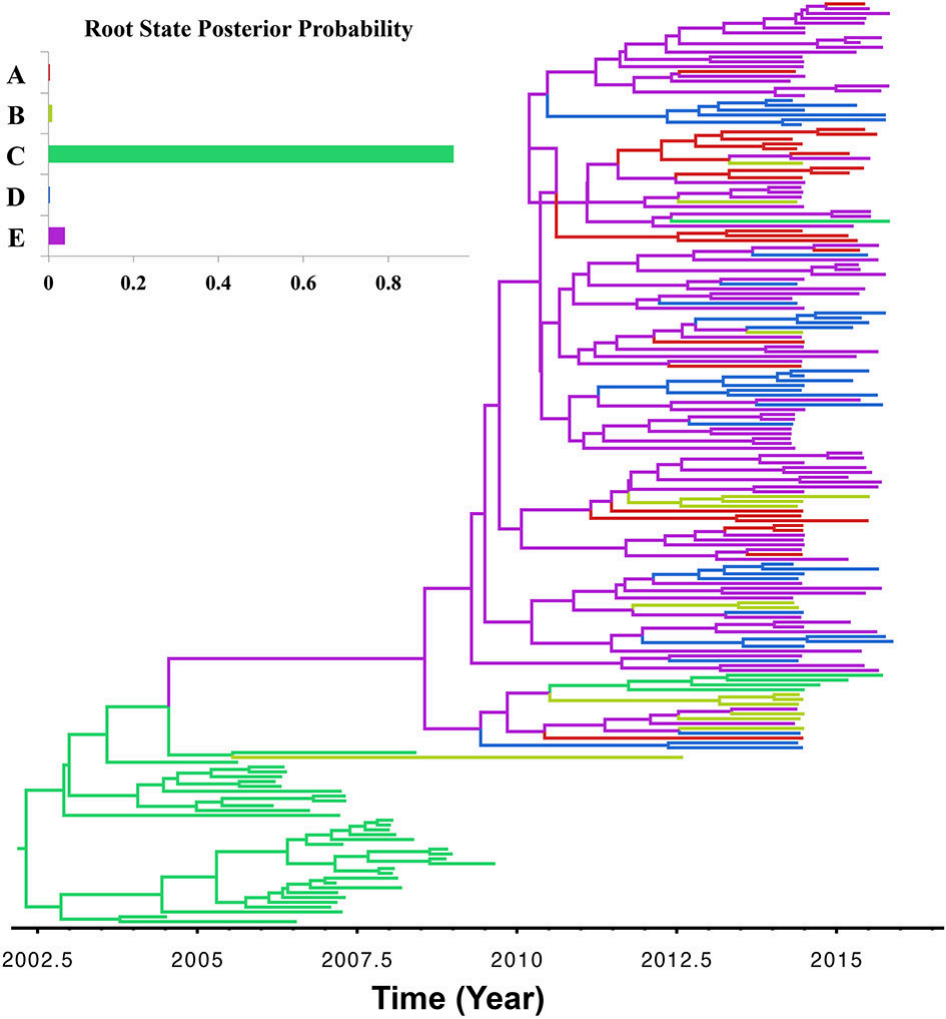
**Maximum clade credibility (MCC) phylogenies of ORF5 gene of Porcine Reproductive and Respiratory Syndrome Virus RFLP type 1-7-4 cluster in the United States**. The color of the branches represents the most probable system type of their descendent nodes. The color-coding corresponds to the upper left figure, which represents the regional system root state posterior probability (RSPP) distributions. The figure was generated from File S6.

**Figure 4:**
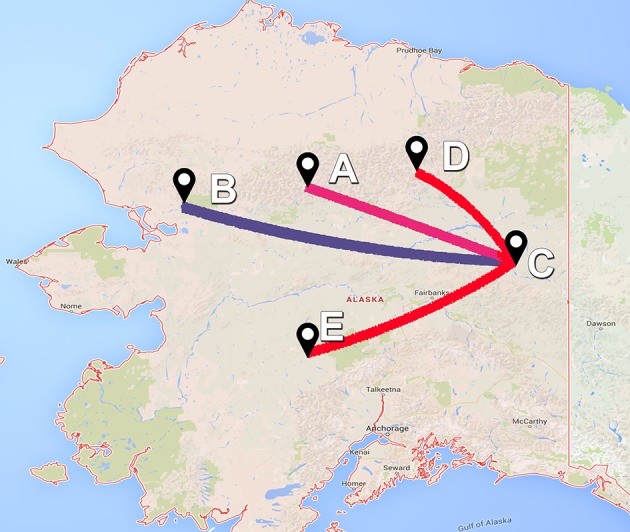
**Bayes factor (BF) test for significant non-zero rates in ORF5 gene of Porcine Reproductive and Respiratory Syndrome Virus RFLP type 1-7-4 cluster in the United States**. Only rates supported by a BF greater than six are indicated. The color of lines correspond to the probability of the inferred transmission rates. Blue and red line gradients indicate relatively weak to strong support, respectively. Site locations for the five systems (A–E) were anonymous and therefore latitude and longitude locations were placed in Alaska.

The asymmetric variants of the discrete-trait model did not achieve full convergence, even after increasing the number of MCMCs to 1 × 10^10^ cycles; and therefore were discarded from the subsequent analyses. Our BF comparisons suggested that the symmetric UCED branch-rate model had the largest log-marginal likelihood (BF > 25), and hence, was chosen as the best fitting phylodynamic model for ORF5 gene regions (Table S2). This result suggested that unidirectional spread of the virus between systems, when designated as origin and destination, had no significant role in the evolution of the currently circulating PRRSV. Figure 3 shows the ORF5 RSPP along with the time-scaled MCC tree (Figure S1). We also generated a KML file to demonstrate the temporal dynamics and spatial diffusion of the virus between systems (File S5). System C was strongly supported as the most likely regional system of origin for the currently circulating RFLP type 1-7-4 with a substantially large RSPP of 0.95. Divergence-time estimates under the discrete trait model indicated that the viral dispersal event from system C was initiated in September of 2000 (95% HPD, July 1999–December 2002). Significant (BF > 6) nonzero rates for the dispersal routes between systems are summarized in Table 2. Our results suggest that the most significant routes of virus exchange were estimated exclusively between system C and all other remaining systems. Interestingly, no significant routes of viral exchange were estimated between systems other than C (Figure 4). Uncertainty and statistical analyses for validating the fit of the sequence data to the selected discrete-trait phylodynamic models are summarized in Table 3. The KL-value suggests that the data under the selected discrete phylodynamic model was able to generate RSPPs that are substantially different from the underlying priors and thus the posterior tree is statistically robust. Furthermore, the AI and PS tests rejected the null hypothesis of no association between sampled system and the structure of the phylogeny (*P* < 0.05). This strongly suggests that the geographical distribution of swine systems are indeed having a significant role in shaping the phylogeny of endemic and newly emerging PRRSV in the US. This role mainly relies on the characteristics of the hog transportation network between systems (Shi et al., 2013; Thanapongtharm et al., 2014; Brar et al., 2015).

**Table 2:** **Bayes factor (BF) tests for non-zero transition rates between system type states**.

**BF**	**Between**	
30529	C	E
30529	C	D
1695	C	A
9	C	B

*BF-values >6 indicate significant rates of directional exchange between systems*.

**Table 3:** **Uncertainty and statistical analyses for assessing the fit of the viral data to the discrete phylodynamic models**.

**Model**	**KL-value^a^**	**Mean values**	**AI^b^**	**95% CI**	***P*-values**	**PS^c^**	**95% CI**	***P*-values**
Systems	2.9	Observed	14.54	(13.28, 15.66)	0.02^*^	124.8	(117.0, 132.0)	0.01^*^
		Null	16.32	(15.09, 17.45)		132.75	(127.70, 136.83)	
Production Type	0.83	Observed	9.9	(8.76, 11.04)	0.61	73.9	(68.0, 80.0)	0.69
		Null	9.63	(8.53, 10.84)		72.7	(68.80, 75.94)	

a *Kullback–Leibler (KL) divergence*.

b *Association index (AI)*.

c *Parsimony score (PS)*.

* *Statistically significant (p-value < 0.05)*.

### Virus transmission patterns in a system

**Figure 5:**
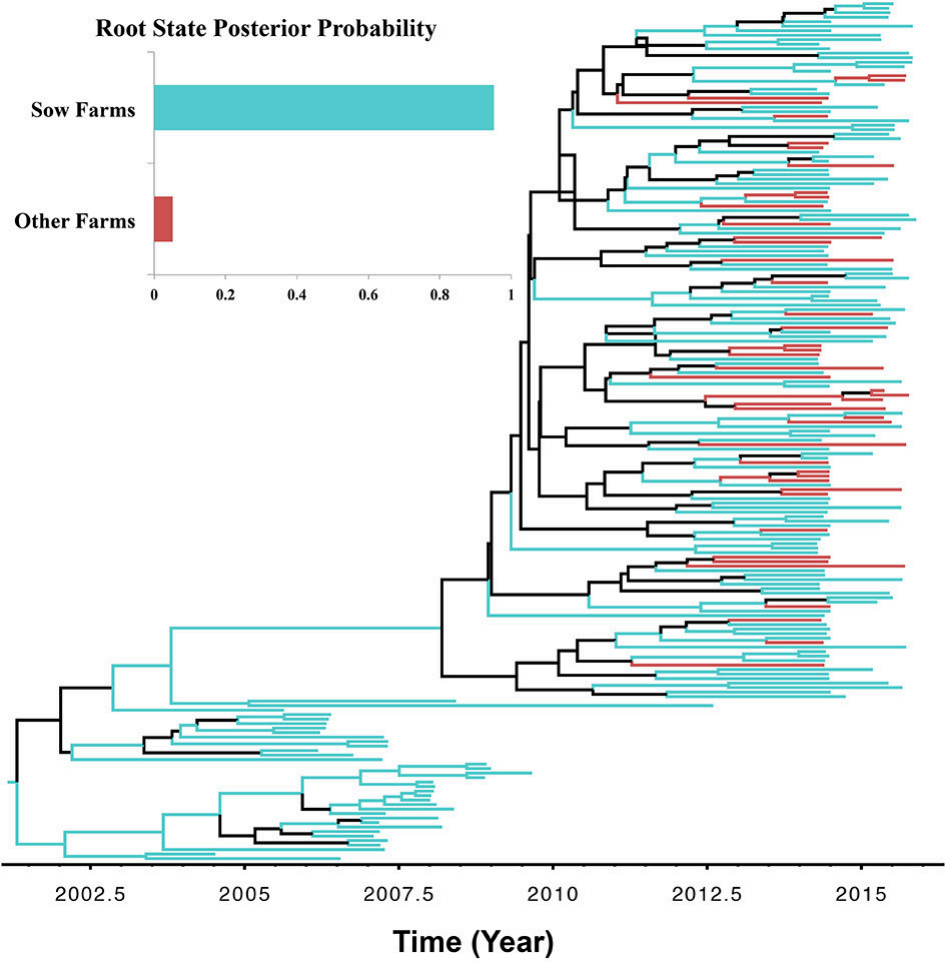
**Maximum clade credibility (MCC) phylogenies of ORF5 gene of Porcine Reproductive and Respiratory Syndrome Virus RFLP type 1-7-4 cluster in the United States**. The color of the branches represents the most probable farm type of their descendent nodes. The color-coding corresponds to the upper left figure, which represents the production type root state posterior probability (RSPP) distributions. Black branches in the tree indicate posterior probability < 0.60. The figure was generated from File S7.

There were two reasons for exploring the role of farm type in viral transmission, (1) to demonstrate how discrete traits may be incorporated in the analysis, and (2) to test the biological soundness of the model results, given that one would expect PRRSV spread to occur mostly from sow farms into other types of farms, following the natural flow of animals. BF comparisons indicated that the Asymmetric UCED branch-rate model with reversible transitions provided the best fit for ORF5 gene regions (Bayes factor > 25 for the log marginal likelihood; Table S2). Sow farms were the most likely ancestral farm type for the currently circulating type 1-7-4 PRRSV (RSPP = 0.95; Figure 5; Figure S2). Our divergence-time estimates suggest PRRSV originated in sow farms approximately in September of 1999 (95% HPD, July 1997–December 2001), and that it was maintained and circulated in sow farms until now. Only one significant nonzero rate transmission route was observed exclusively (BF > 6) from sow farms to all other farms. However, most branches of the MCC tree under the farm type phylodynamic model were weakly supported (Branch rate posterior probability < 0.6). In addition, the low KL-value under the farm type model was substantially less robust, when compared to the systems model (Table 3). This is because, small KL divergence statistic values between any prior and posterior probability distributions indicate that the data contain little information regarding the value of the selected parameter, and therefore, its posterior probability distribution will be similar to the corresponding prior probability distribution (Lemey et al., 2009). Furthermore, the AI and PS test failed to reject the null hypothesis of no association between farm type and the structure of the phylogeny (*P* > 0.05). This is expected because the typical structure of swine farms in the US is farrow-to-weaning, which in turn segregates breeding pigs from growing pigs, and thus, makes sow farms more likely as sources of virus spread through pig movement than growing pigs sites from which most pigs go to market (Jeong et al., 2014). Therefore, and as suggested elsewhere, sow farms are more likely sources of transmission and maintenance of newly emerging PRRSVs (Kwong et al., 2013).

### Value to participants, the swine industry, and society

The veterinarians and pork producers who voluntarily share the disease status and location of their farms are vanguards in food production. By doing so, the individual participant or a particular farm risks being identified, either correctly, or incorrectly, as being a source of virus evolution and spread to other farms. And yet, the nature and structure of the swine industry is much more responsible for pathogen movement than any individual farm. That is, weaned pigs are transported away from the sow farm to allow pathogens such as PRRSV to be more effectively eliminated from the sow herd. This means that growing pigs must be transported to nursery and finishing sites and by doing so, pathogens are also conveniently moved around the country. Secondly, health is difficult to maintain if growing pig sites become too large. Therefore, we have a distributed system of growing pig sites which also lends to pathogens being moved around the country. Finally, farms might be using live virus vaccination in the short term to reduce clinical impact and aid in the elimination of field virus in the long term. So it is a bold decision to share data for a study such as this. There is a greater good being pursued by these industry leaders. They voluntarily share their premises identities and pathogen status in the interests of national disease control such that we might detect emerging pathogens earlier than otherwise and take actions accordingly. Work such as reported in this paper is on the cusp of a new era of disease control.

### Considerations and future applications of the method

The methodological approach presented here entailed several compromises, including: (1) imprecise epidemiological information related to the discrete traits investigated, and (2) incomplete and biased sampling of PRRSV ORF5 sequences. For the first, we demonstrate the impact of the accuracy and availability of epidemiological information on the MCC trees (Figures 3, 5) and their posterior inferences. This impact on the performance of phylodynamic models has been discussed elsewhere (Chaikhumwang et al., 2015). However, this issue is chronic in the context of surveillance data and almost impossible to avoid in practical reality of animal disease surveillance (Perez et al., 2011). Therefore, rigorous analysis of a selected Bayesian phylodynamic model (i.e., assessing fit and uncertainty) is essential before deriving conclusions from their posterior inferences. For the second, inferences under the phylodynamic models assume that we have either a complete or random sample of sequence data. In the present case, this requires that the PRRSV sequences were collected randomly with respect to time (between 1999 and 2015) with their corresponding epidemiological information. Like most phylogenetic studies, our data were from a convenience sample and might suffer from strongly biased samples. The impact of these departures from random sampling on the estimates is difficult to quantify (Alkhamis et al., 2015). However, our study is based on all available sequence data from our participants for the ORF5 gene associated with the currently circulating RFLP type 1-7-4 epidemic in the US, and therefore reflects our best understanding based of the available data. It is worth noting that despite the unequal number of sequences obtained from different systems (Table S1), our posterior inferences for dispersal of the virus between system was not biased toward systems with more included sequences, such as E (*n* = 120) and D (*n* = 55), when compared to C (*n* = 52). This constitutes an example for the utility and robustness of such methods in the context of molecular surveillance of swine diseases.

Bayesian phylodynamic models have not yet been widely accepted as a resource by veterinary agencies to support disease surveillance, control and prevention strategies. This is attributed by part to the intensive computational requirements of the methods presented here. For example, we were unable to assess the topology of 6774 sequences using BEAST due to the lack of sufficient computational resources. Instead, we used the traditional ML method to help in identifying the key cluster of interest, while reducing the computational requirements of the Bayesian analyses used to address our main hypotheses. That said, computational resources are in continuous improvement in terms of speed and cost, and therefore, in the near future the presented analytical pipeline can be completely transformed to Bayesian statistical framework. However, previous use of such methods on avian influenza and the Ebola epidemics demonstrated the ability of phylodynamic methods to shed novel insights into the evolutionary epidemiology of infectious diseases and provide support for decisions regarding animal and public health (Lam et al., 2012; Pybus et al., 2013; Alizon et al., 2014). Our phylodynamic analyses of a PRRSV ORF5 sequence dataset and associated epidemiological information, in an endemic country like the U.S., were in agreement with previous inferences about the demographic histories and population growth patterns of viral lineages and sub-lineages of the virus in the U.S. (Shi et al., 2010). Bayesian phylodynamic models show one remarkable improvement compared to traditional methods, namely, they make use of associated epidemiological information, such as time and place of isolation, to infer genetic relations. The inclusion of information on nucleotide substitution schemes obtained from the data, allowing for different model assumptions to assess the degree of genetic relatedness under time-scaled phylogenies, has provided a robust strategy, for example, to distinguish between potentially related PRRSV strains detected in air samples and swine farms in high and low swine density regions (Brito et al., 2014). In the analysis here, we incorporated time of prior isolation to reconstruct the phylogenetic dendogram, hence, making use of temporal distances to infer genetic relations. This approach can help to shed further light on several evolutionary and epidemiological characters of endemic PRRSV. Furthermore, extended phylodynamic models can provide insights on the ancestral origins of the outbreak between swine systems (e.g., the ancestral system or herd type) and spatio-temporal progression of an epidemic. These inferences could be used, for example, to identify viral dispersion routes that correspond with transportation patterns involving high PRRSV risk.

## Conclusion

Classical phylogenetic methods such as neighbor-joining or maximum likelihood trees, provide limited inferences about the evolution of important pathogens and ignore important evolutionary parameters and uncertainties, which in turn limits decision making related to surveillance, control, and prevention resources. However, in this study, we illustrated the applications and potential of phylodynamic methods as tools for molecular surveillance of food animal viruses by assessing the evolution of newly emerging PRRSVs in the U.S. We analyzed different epidemiological and evolutionary aspects of a recently collected ORF5 gene sequence dataset. Using coalescence and discrete trait phylodynamic models, we obtained a phylogeny adjusted for many important epidemiological parameters such as space, time, and host type. Furthermore, we were able to (1) infer population growth and demographic history of the virus, which aids in assessing the magnitude of epidemic progression; (2) identified the most likely ancestral system, which aids in guiding risk-based surveillance activities; and (3) modeled viral transmission patterns between systems and farm types, which sheds important insights about viral transmission dynamics between and within swine herds. Accordingly, incorporating phylodynamic analyses as a standard tool for the molecular surveillance of swine diseases might support the development of more effective economically rational policy decisions for the control of PRRSV in high-risk systems. However, investments must be mobilized toward improving genomic databases and building efficient bioinformatics and computational infrastructures, which are the base requirements for the field of applied phylodynamics (Scotch et al., 2011; Scotch and Mei, 2013).

## Author contributions

MA formulated the Bayesian models and was primarily responsible for report and manuscript preparation; AP provided interpretation on the use of epidemiological models, collaborated in the design of the analytical model, and assisted in manuscript preparation; MM contributed with the interpretation of results related with PRRSV genetic dynamics and manuscript preparation and editing; XW helped with data preparation and management; RM conceived the study, was responsible for communication with the industry and supervision of the entire project, provided insight on the implementation of results at the field level, and assisted in manuscript preparation.

### Conflict of interest statement

The authors declare that the research was conducted in the absence of any commercial or financial relationships that could be construed as a potential conflict of interest.
