# Supplementary material for: Applications of Bayesian Phylodynamic Methods in a Recent U.S. Porcine Reproductive and Respiratory Syndrome Virus Outbreak
Source: Front Microbiol. 2016 Feb 2;7:67. doi: 10.3389/fmicb.2016.00067 (PMC4735353; doi:10.3389/fmicb.2016.00067)
Supplement: Supplementary file 1 [file DataSheet1.DOCX]

**Applications of Bayesian Phylodynamic Methods in a Recent U.S. Porcine Reproductive and Respiratory Syndrome Virus Outbreak**

Mohammad A. Alkhamis *^1,2^, Andres M. Perez ^1^, Michael P. Murtaugh^3^, Xiong Wang^1,3^, Robert B. Morrison^1^

*** Correspondence:** Mohammad A. Alkhamis. malkahmi@umn.edu

1. **Supplementary Data**

**File S1. Tree file for the maximum likelihood phylogenies of ORF5 gene of Porcine Reproductive and Respiratory Syndrome Virus RFLP type 1-7-4 cluster in the United States.**

**File S2. Population size XML.** XML file used to infer Bayesian time-scaled phylogeny and estimate effective population size through time (Figure 2) for the entire dataset of 288 PRRSV RFLP type 1-7-4 ORF5 sequences. The file comprises the multi-sequence alignments and all BEAST parameters used in this study.

**File S3. Dispersal between systems XML.** XML file used to infer Bayesian time-scaled phylogeny and estimate viral dispersal history between regional systems (Figure 3 and 4; Table 2) for the entire dataset of 288 PRRSV RFLP type 1-7-4 ORF5 sequences. The file comprises the multi-sequence alignments and all BEAST parameters used in this study.

**File S4. Transmission in a system XML.** XML file used to infer Bayesian time-scaled phylogeny and model viral transmission in a systems (Figure 5) for the entire dataset of 288 PRRSV RFLP type 1-7-4 ORF5 sequences. The file comprises the multi-sequence alignments and all BEAST parameters used in this study.

**File S5. Temporal dynamics of the spatial diffusion of Porcine Reproductive and Respiratory Syndrome Virus RFLP type 1-7-4.** The KML file demonstrates the dispersal pattern of PRRSV between 2003 and 2015. Lines between locations represent branches in the MCC tree along which spatial transmission occurs. The diameters of circles are proportional to square root of the number of MCC branches maintaining a particular location state at each time-point. The blue and red color gradients reflect the relative age of the transitions for ORF5 (older-recent, respectively).

**File S6. Tree file for the maximum clade credibility (MCC) phylogenies of ORF5 gene of Porcine Reproductive and Respiratory Syndrome Virus RFLP type 1-7-4 cluster in the United States.**

**File S7. Tree file for the maximum clade credibility (MCC) phylogenies of ORF5 gene of Porcine Reproductive and Respiratory Syndrome Virus RFLP type 1-7-4 cluster in the United States.**

1. **Supplementary Figures and Tables**

## Supplementary Figures


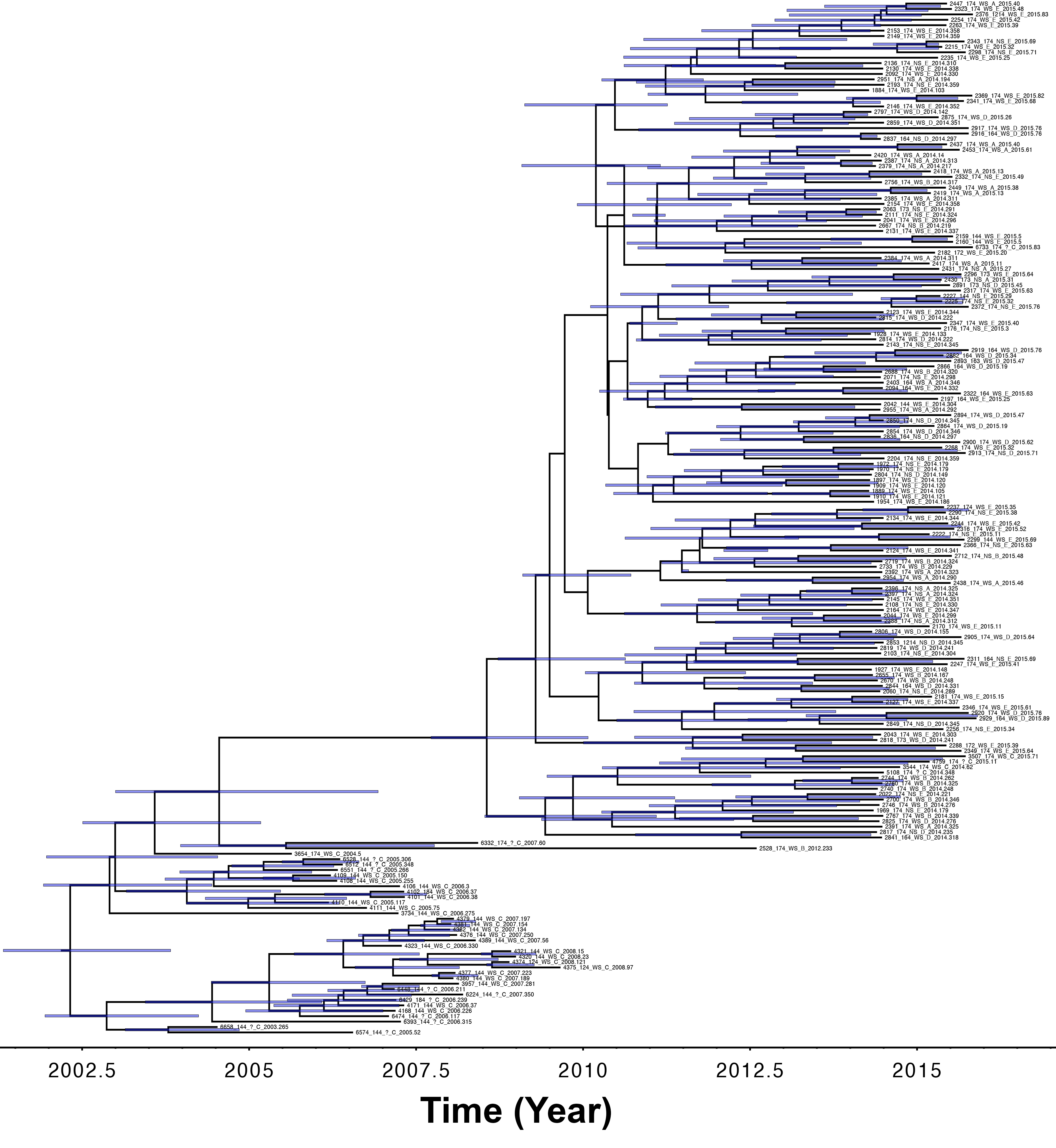


**Figure S1.** **Maximum clade credibility (MCC) trees of Porcine Reproductive and Respiratory Syndrome Virus RFLP type 1-7-4 cluster.** Branch lengths are rendered proportional to absolute time (see timescales). Nodes correspond to median ages and the blue horizontal bars at nodes represent the corresponding 95% HPDs for divergence-time estimates. The tree is complementary to Figure 3.


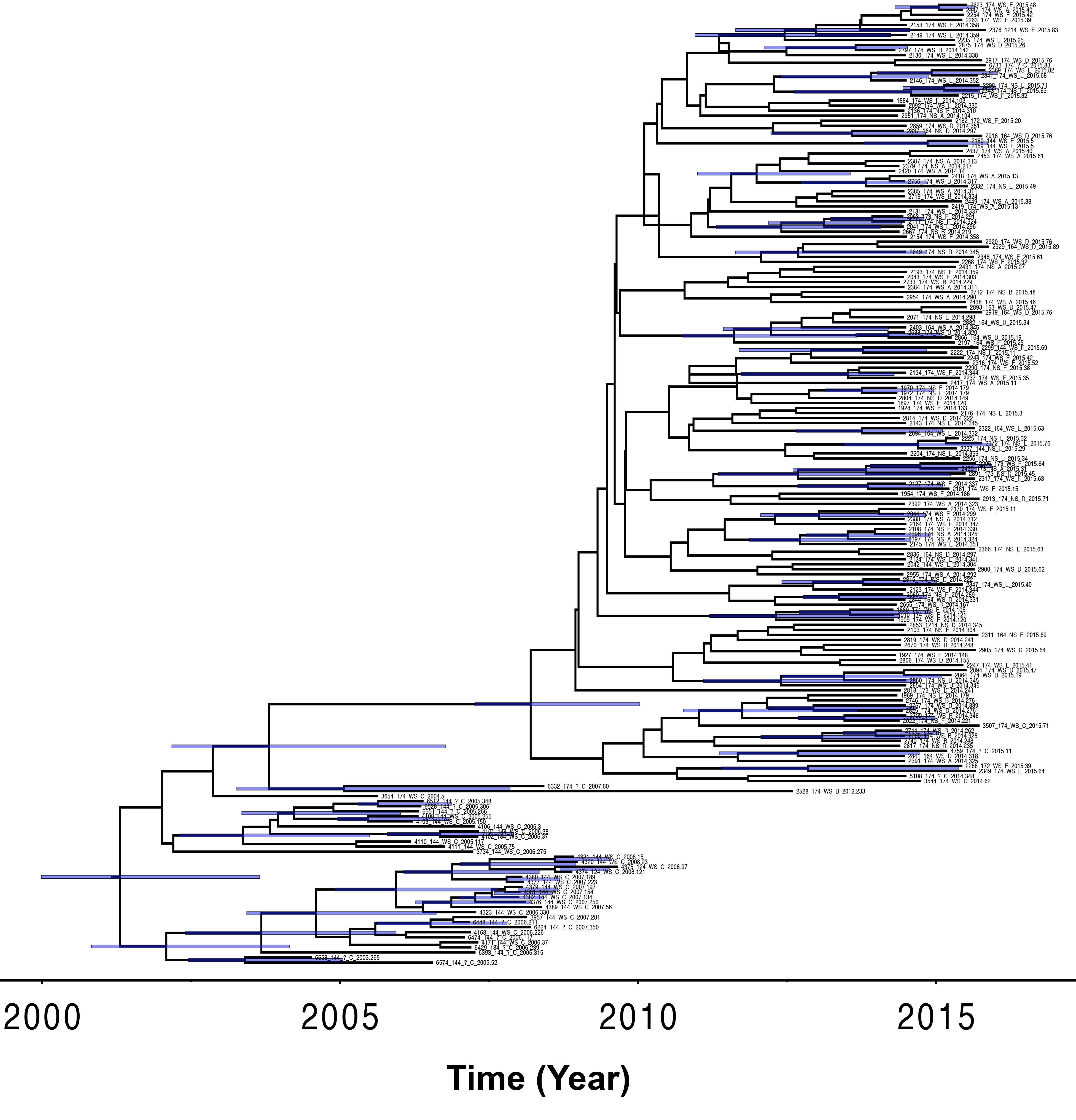


**Figure S2.** **Maximum clade credibility (MCC) trees for Porcine Reproductive and Respiratory Syndrome Virus RFLP type 1-7-4.** Branch lengths are rendered proportional to absolute time (see timescales). Nodes correspond to median ages and the blue horizontal bars at nodes represent the corresponding 95% HPDs for divergence-time estimates. The tree is complementary to Figure 5.

## Supplementary Tables

**Table S1.** Summary profile of PRRSV sequences isolated in the United States between September 2003 and March 2015 (*N* = 288).

| System | N. Sequences | Host type | N. Sequences | Collection Dates |
| --- | --- | --- | --- | --- |
| A | 43 | Other farms | 10 | Jan-2014 to Mar-2015 |
|  |  | Sow farms | 33 |  |
| B | 18 | Other farms | 3 | Aug-2012 to Feb-2015 |
|  |  | Sow farms | 15 |  |
| C | 52 | Unknown* | 16 | Sep-2003 to Mar-2015 |
|  |  | Sow farms | 36 |  |
| D | 55 | Other farms | 15 | May-2014 to Mar-2015 |
|  |  | Sow farms | 40 |  |
| E | 120 | Other farms | 35 | Apr-2014 to Mar-2015 |
|  |  | Sow farms | 85 |  |

*Production type information is unknown

**Table S2.** Candidate phylogeographic models for ORF5 gene explored for relative fit using AICM. Best models are highlighted with boldface.

| BEAST analysis | Model | Site | Discrete traits | Branch rates | Trees | AICM (SE) |
| --- | --- | --- | --- | --- | --- | --- |
| *Population size* | 1 | GTR+GAMMA | NA^a^ | UCLN | Coalescent: Bayesian Skyline | 8954 (0.64) |
|  | **2** |  |  | **UCED** |  | **8870 (0.59)** |
| *Divergence time and growth rate* | 1 |  | NA^a^ | UCLN | Coalescent: Constant Size | 8956 (1.30) |
|  | 2 |  |  | UCED |  | 8897 (0.66) |
|  | 3 |  |  | UCLN | Coalescent: Exponential Growth | 8948 (0.75) |
|  | 4 |  |  | UCED |  | 8894 (1.15) |
|  | 5 |  |  | UCLN | Coalescent: Expansion Growth | 8901 (0.76) |
|  | **6** |  |  | **UCED** |  | **8891 (0.52)** |
|  | 7 |  |  | UCLN | Coalescent: Logistic Growth | 8911 (1.2) |
|  | 8 |  |  | UCED |  | 8955 (0.82) |
| *Host type* | 1 |  | Symmetric reversible | UCLN | Coalescent: GMRF Bayesian Skyride | 9298 (0.51) |
|  | 2 |  |  | UCED |  | 9177 (1.03) |
|  | 3 |  | Asymmetric irreversible | UCLN |  | 8934 (0.88) |
|  | **4** |  |  | **UCED** |  | **8840 (1.61)** |
| *System type* | 1 |  | Symmetric reversible | UCLN |  | 9450 (0.51) |
|  | **2** |  |  | **UCED** |  | **9334 (0.79)** |
|  | 3 |  | Asymmetric irreversible | UCLN |  | NC* |
|  | 4 |  |  | UCED |  | NC* |

***** Full model Convergence failed after 1x10^10^ MCMC cycles.

^a^ Not applicable
